# Learning Modulates Early Encephalographic Responses to Distracting Stimuli: A Combined SSVEP and ERP Study

**DOI:** 10.1523/JNEUROSCI.1973-24.2025

**Published:** 2025-04-04

**Authors:** Dock H. Duncan, Norman Forschack, Dirk van Moorselaar, Matthias M. Müller, Jan Theeuwes

**Affiliations:** ^1^ Department of Experimental and Applied Psychology, Vrije Universiteit Amsterdam, 1081 HV Amsterdam, The Netherlands; ^2^Institute for Brain and Behavior Amsterdam (iBBA), 1081 HV Amsterdam, The Netherlands; ^3^Wilhelm Wundt Institute for Psychology, University of Leipzig, 04109 Leipzig, Germany; ^4^William James Center for Research, ISPA-Instituto Universitario, 1149-041 Lisbon, Portugal

**Keywords:** attention, distraction, ERP, Pd, SSVEP, alpha, statistical learning

## Abstract

Through experience, humans can learn to suppress locations that frequently contain distracting stimuli. However, the neural mechanism underlying learned suppression remains largely unknown. In this study, we combined steady-state visually evoked potentials (SSVEPs) with event-related potentials (ERPs) to investigate the mechanism behind statistically learned spatial suppression. Twenty-four male and female human participants performed a version of the additional singleton search task in which one location contained a distractor stimulus frequently. The search stimuli constantly flickered on-and-off the screen, resulting in steady-state entrainment. Prior to search onset, no differences in the SSVEP response were found, though a post hoc analysis did reveal proactive alpha lateralization. Following search onset, clear evoked differences in both the SSVEP and ERP signals emerged at the suppressed location relative to all other locations. Crucially, the early timing of these evoked modulations suggests that learned distractor suppression occurs at the initial stages of visual processing.

## Significance Statement

Often times a distractor (e.g., a colorful yet irrelevant billboard on a highway) becomes easier to ignore after you have encountered it several times. This learned suppression is an important component of the human visual system which is otherwise highly salience driven. In a series of EEG experiments, we used steady-state visually evoked potentials and event-related potentials to study how learning changes attention toward salient distracting stimuli when these distractors appear frequently at specific locations. We found converging evidence that learning alters the early evoked responses to these stimuli. Our results indicate that through learning, early neural responses to distracting stimuli are changed.

## Introduction

The world is inherently distracting as at any moment, most perceptual input is irrelevant to our current goals. As such, the ability to ignore distracting information is crucial for cognitive functioning. A key feature of the environment that facilitates the filtering of irrelevant perceptual input is that distracting stimuli frequently appear in predictable patterns and at predictable locations. These regularities allow for statistical learning—a fundamental aspect of cognition (Friston, 2010; Turk-Browne, 2012; Frost et al., 2019)—to aid in distractor rejection (Theeuwes et al., 2022). Recent research has focused on how these learned regularities affect attentional processing, emphasizing the role of statistical learning in conjunction with top-down and bottom-up forms of attention in shaping attentional orienting (Awh et al., 2012; Anderson et al., 2021; Theeuwes, 2025).

While it is well established that salient distractors can capture attention involuntarily (Theeuwes, 2010), recent research has demonstrated that such interference can be attenuated through learning about probable distractor locations (Ferrante et al., 2018; Wang and Theeuwes, 2018) and features (Vatterott and Vecera, 2012; Cunningham and Egeth, 2016; Kim et al., 2023). However, the exact mechanisms underlying this learned suppression and its temporal dynamics are still debated. As outlined by Liesefeld et al. (2024), suppression can occur at three distinct moments in time: (1) before the distractor appears, reflecting anticipatory preparation; (2) after distractor onset but before attentional capture, indicating rapid early filtering; or (3) after attentional capture has occurred. A central debate concerns whether suppression operates proactively—before attention is deployed (Scenarios a and b)—or reactively, following attentional capture (Scenario c). Behavioral studies have provided evidence for both proactive (Kong et al., 2020; Huang et al., 2021, 2022, 2023; Ivanov and Theeuwes, 2021; Duncan et al., 2024; van Moorselaar and Theeuwes, 2024) and reactive mechanisms (Addleman et al., 2019; Di Caro et al., 2019; Wang et al., 2019a; Sauter et al., 2021; Chang et al., 2023). Similarly, neuroimaging work has yielded support for both proactive (Wang et al., 2019b; Zhang et al., 2022; Duncan et al., 2023b; Richter et al., 2024) and reactive accounts (Huang et al., 2025), leaving it unclear whether statistical learning prevents distractor processing entirely, attenuates initial processing, or merely speeds the disengagement of attention from capture.

The majority of neural studies on statistically learned distractor suppression have utilized EEG (Gaspelin and Luck, 2018; van Moorselaar and Slagter, 2019; Wang et al., 2019b; van Moorselaar et al., 2020, 2021, 2023; Stilwell et al., 2022; Hanne et al., 2024), with many studies focusing on a lateralized positive deflection in posterior electrodes, labeled the P_D_, that is strongly associated with distractor processing (Hickey et al., 2009; Gaspelin et al., 2023). While these studies have relied on variations in P_D_ latency to indirectly distinguish between proactive and reactive mechanisms (Gaspelin et al., 2023), recent work has provided direct evidence of proactive suppression through presearch modulations in tagged frequency responses—enabling real-time observation of anticipatory processing (Ferrante et al., 2023). Even though in this study, the techniques used to analyze the steady-state visual evoked potentials (SSVEPs) prevented a time course analysis, techniques analyzing the time course of slower steady states have already extensively verified its excellent temporal fidelity (Müller et al., 1998; Andersen et al., 2011; Norcia et al., 2015) and these SSVEPs have previously been used to study the time course of distraction with temporal precision (Forschack et al., 2022a,b). Combining this SSVEP-based temporal analysis with traditional ERPs thus has the potential to not only better understand the mechanisms underlying learned distractor suppression but also to illuminate the origin of the observed latency variability across P_D_ studies (Gaspelin et al., 2023).

The current study used SSVEPs in a novel version of the additional singleton task (Theeuwes, 1992). Participants searched for a unique shape while ignoring a salient distractor, which appeared more frequently at one location in space to induce statistically learned distractor suppression (Wang and Theeuwes, 2018). Critically, search stimuli flickered at individual frequencies, enabling EEG SSVEP measurements. By examining SSVEP modulation prior to and following search display onset in combination with well-known reactive ERP components, we aim to elucidate how attention orients toward distractors at high- versus low-probability locations. This approach allows us to measure both proactive and reactive indices of suppression, providing a comprehensive view of the temporal dynamics of learned suppression.

## Materials and Methods

### Participants

The experiment was conducted at the Brain and Behavior Labs on the campus of Vrije Universiteit Amsterdam and was approved by the Ethical Review Committee of the Faculty of Behavioral and Movement Sciences and conformed to the Declaration of Helsinki. All participants gave informed consent prior to participation in the study and were compensated with 50 euros or course credits for a total of ∼4 h of participation across two experimental sessions. All participants indicated normal or corrected-to-normal vision and no history of cognitive impairments. Based on the effect size reported in Ferrante et al. (2023) for their statistical learning effect (within subject *η*^2^ = 0.27) and also to accommodate our counterbalanced design, a final sample of 24 participants (16 female, median age 20, sensitive to within-subject *η*^2^ > 0.175 with power = 0.95 and alpha = 0.05) were recruited after replacement of five participants. One participant was excluded for excessive eye movements, two for low trial counts after data cleaning, and two for technical issues during data recording. This sample size roughly matches those used in other similar studies using SSVEPs (Andersen and Müller, 2010; Forschack et al., 2017; Moratti et al., 2024).

### Experimental setup

Participants were seated in a dimly lit, sound-attenuated room, with their heads in a chinrest 70 cm away from a 526 × 296 mm (1,920 × 1,080 pixel) ASUS ROG STRIX XG248 LED monitor with a refresh rate of 240 Hz. The task was programmed in Matlab R2020a (The MathWorks) using functions from the Psychophysics Toolbox 3 (Kleiner et al., 2007) and custom scripts run on a Windows operating system (Windows 10). EEG data was recorded with default Biosemi settings at a sampling rate of 512 Hz using a 64-electrode cap with electrodes placed according to the international 10–10 system (Biosemi ActiveTwo system; Biosemi.com) with two reference electrodes attached to the mastoids. Vertical (VEOG) and horizontal EOG (HEOG) were recorded via electrodes placed ∼2 cm above and below either the left or right eye and two electrodes placed ∼1 cm to the left and right of the outer canthi. Eye tracking data was additionally collected using an EyeLink 1000 (SR Research) calibrated at the beginning of each experimental quarter and used to live monitor participants fixation.

Participants performed a version of the additional singleton task (Theeuwes, 1992) with an underlying distractor spatial regularity—a paradigm known to cause statistical distractor learning (Wang and Theeuwes, 2018). All stimuli were presented on a black background. The fixation point (∼0.35°) was a circular shape with an embedded cross hair, a combination that has been shown to best enforce stable fixation (Thaler et al., 2013). In between search displays, search locations were occupied by placeholder shapes, created by overlaying a diamond over a gray circle, which continuously flickered to maintain the steady-state entrainment (described below). Search displays consisted of eight equally spaced shapes presented on an imaginary circle centered at fixation (radius, ∼6°). Search shapes could either be diamonds (∼2.6° × 2.6° square rotated 45°) or circles (radius, ∼1.5). The color of the search shapes was determined per participant via a pre-experiment isoluminant adjustment task whereby the perceptual luminance of green and orange was matched to the luminance of the gray placeholders (RGB 115, 115, 115) using heterochromatic flicker photometry (Wagner and Boynton, 1972). All shapes also contained a horizontally or vertically oriented black line (∼1°, RGB 0,0,0, four vertical and four horizontal on every trial). The displays were rendered such that each display contained one unique shape (i.e., the target), and on a subset of search trials, one of the homogeneous shapes had a unique color (the singleton distractor).

**Figure 1.**
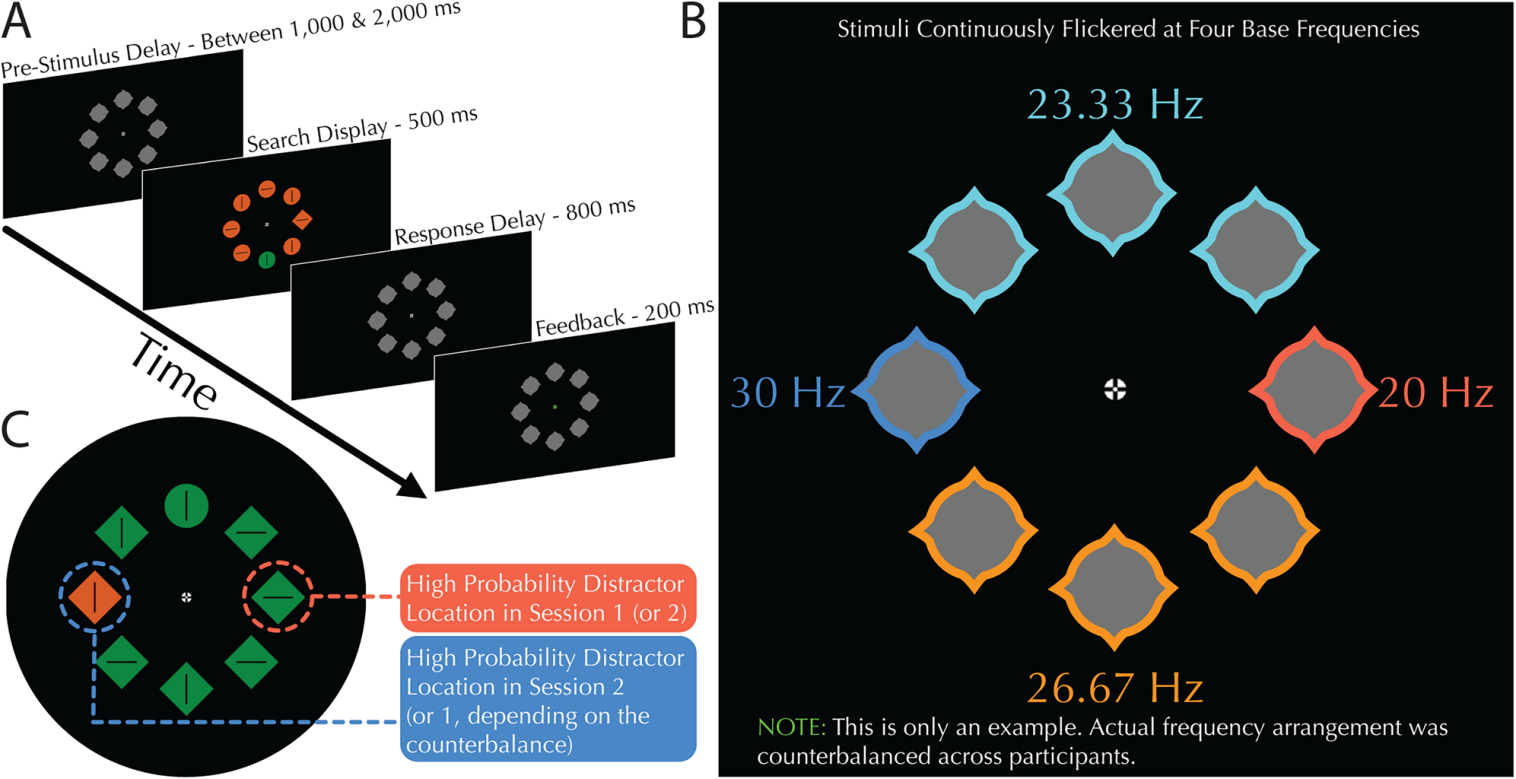
Behavioral paradigm and stimuli. ***A***, Trial breakdown including presentation times of the various screens. Interstimulus intervals were jittered such that they randomly varied between 1,000 and 2,000 ms in duration. Note that search stimuli only appeared at the moment that the four frequencies aligned. Participants were able to respond in a time window of 1,300 ms after search display onset. ***B***, Example of the gray placeholder stimuli with different tagging frequencies represented as different color outlines for illustration that stimuli of the same color flickered at the same frequency (colors were not presented during the experiment). The top three and bottom three stimuli flickered at their own respective frequencies while the far left and far right stimuli had unique frequencies, respectively. The actual distribution of frequencies was counterbalanced across participants such that every combination of the four frequencies was used once in the 24-participant dataset. ***C***, Example search display with high-probability and low-probability location highlighted. Each participant completed two sessions, one with a high-probability distractor location on the right side of the screen and one with it on the left side of the screen (order counterbalanced). Response times tagged responses and ERPs were all restricted to trials in which distractors or targets appeared at these two horizontal locations.

All eight shapes (placeholders and search items) flickered in an on/off mode at a certain frequency synchronized to the screen refresh rate through the course of each experimental block. Four fundamental frequencies were used (20, 23.33, 26.66 and 30 Hz) based on multiples of the base rate of 3.33 Hz which was calculated as a viable subdivision given the refresh rate of 240 Hz. The eight shapes were separated into four groups with different flickering frequencies—the top three and bottom three shapes each flickered at their own frequency, while the far left and right shapes flickered at their own frequencies (Fig. 1*B*). The flicker frequencies of the top, bottom left, and right of the screen were counterbalanced across participants, with each of the 24 participants having a different arrangement of frequencies which stayed the same across both experimental sessions. The flicker presentation utilized a square wave, as this has been shown to better engender steady states than sinusoidal waves (Panitz et al., 2023).

### Experimental design

Participants were tasked with finding a unique shape in a display of eight shapes arranged in a ring around fixation (the additional singleton task; Theeuwes, 1992; Fig. 1*C*). The shape target was either a circle or a diamond, swapping randomly across displays. Participants’ task was to wait until a search display to onset, identify the shape singleton in the search array, and report whether the black line within this shape was oriented vertically (by pressing the “up” arrow button) or horizontally (by pressing the “left” arrow button on a standard QWERTZ keyboard). Although search displays were embedded in a continuous stream of visual stimulation (to ensure continuation of the steady state in the encephalographic signal), we refer to each onset of search and the corresponding response as “search trials.” Each search trial was preceded by a jittered intersearch interval (1,000 and 2,000 ms) where the neutral placeholders would flicker. The search stimuli would then appear on the screen for 500 ms. Search onset was timed such that they would appear at the moment the four frequencies synchronized, thereby ensuring that immediately before the eight search stimuli appeared on screen, the screen would be blank. Following search stimuli offset, participants would be given 800 additional milliseconds to provide a response, making a total response window of 1,300 ms.

On the majority of search displays (80%) a unique color singleton distractor was presented. This distractor was always presented in the opposite color as the target (either green or orange). To reduce the variation in search conditions for analysis, targets and distractors were only ever presented on the four cardinal locations (top, bottom, left and right) with the diagonal locations only ever containing target-color matching nontargets. Because our analyses were primarily interested in trials in which a distractor was present on a horizontal position, and the targets at a vertical position, this design choice greatly reduced the number of superfluous trials collected. Previous work has shown that this does not change the effects of attentional capture at the behavioral (Jungerius et al., 2024) or encephalographic level (van Moorselaar et al., 2023), thereby making it a choice that greatly reducing experiment time at the sacrifice of introducing a peripheral task regularity with little consequence. Critically, one of the two horizontal locations was selected to be the high-probability (HP) distractor location and was three times more likely to contain a distractor than the other three low-probability (LP) locations (50% of all distractor trials; 540 HP trials and 180 LP trials per LP location per session). Also, the target distribution was made approximately equal across the four locations, with targets ∼1.3 times more likely to appear in one of the distractor low-probability locations (280 targets at the HP location vs 357 targets at each of the three remaining LP location per session).

The experiment was split into two separate sessions occurring on separate days (scheduled between 1 d and 1 week apart in time). Each session lasted ∼2 h including EEG capping. Session one began with a comprehensive written description of the task and experimental flow, including visual examples of the stimuli. Next, participants started a practice block of 30 search trials which they could repeat as many times as needed to understand the task before the real experiment started. Participants next performed 30 blocks of 45 search trials (∼3 min per block). Breaks were offered between each block where participants were informed of their accuracy and average reaction time and were encouraged to rest their eyes if needed before continuing. Once an experimental block began, the placeholders would begin to flicker at their underlying frequencies and stimuli at these locations would continue to flicker for the entire block without stopping, including when placeholders swapped to search stimuli where the search stimuli would carry on the flickering. Following the collection of seven subjects (and in response to participant feedback), trial feedback was incorporated into the experimental design. This would take the form of the fixation dot either turning red or green for 200 ms, depending on whether the previous search trial was correct or incorrect. A subset of participants (*n* = 7) did not receive trial feedback and instead had a longer window in which to provide a response.

Following the completion of session two, participants got a verbal debrief in which they were asked whether they noticed that the targets and distractors were only present on the four cardinal locations. They were next asked if they noticed the distractor regularity and were asked to guess where they thought the distractor was most likely to occur on the last block they had completed. If a participant both indicated that they noticed the distractor regularity and were able to report the distractor high-probability location on the previous block, then they were labeled as aware of the regularity.

### Behavioral preprocessing

Participants’ behavioral data was collapsed across the two sessions. Because learning effects necessarily require some experience to manifest, the first 100 search trials of each experimental session were excluded from analysis. All search trials with RTs below 200 ms were excluded. All incorrect search trials (∼14%) and trials in which the reaction time was 2.5 standard deviations away from that participant's average reaction time (∼4%), were excluded from behavioral analysis.

### EEG preprocessing

Similar to our behavioral analysis, the first 100 search trials of each experimental session were excluded from analysis to better isolate responses after learning had occurred. Analysis was further restricted to correct search trials. Functions from the EEGLAB toolbox (Delorme and Makeig, 2004) along with custom MATLAB scripts (The MathWorks) were used to prepare the EEG data. Much of the preprocessing pipeline was a direct adaptation of that used in the work of Forschack et al. (2022a,b). In a first step, the continuous data were processed through the standardized early-stage EEG processing pipeline (PREP v0.55.3; Bigdely-Shamlo et al., 2015). This algorithm first removed 50 Hz line noise by subtracting a frequency domain regression model of the best-fitting deterministic sinusoid in the range of 48–52 Hz estimated by a sliding window multitaper approach. Then, the algorithm rereferenced the continuous data to a robust average reference signal derived by iteratively detecting and interpolating noisy channels (interpolation based on all electrodes excluding EOGs, note this is preferred to mastoid rereferencing in PREP; Bigdely-Shamlo et al., 2015). Next, to identify and remove ocular, muscular, and other non-neural artifacts from the EEG signal, individual session datasets underwent independent component analysis (ICA) using the adaptive mixture of independent component analyzers (AMICA) package by Palmer et al. (2012). For this, training datasets (concatenated blocks) for the ICA were preprocessed by a high-pass filter of 1 Hz. For training the ICA, contiguous epochs of 1 s were extracted from which the average epoch potential was subtracted. These ICA epochs were screened for nonstereotypical artifacts and rejected if contaminated. Rather than manually selecting ICA components for exclusion, the ICLabel classifier (Pion-Tonachini et al., 2019) was used to automatically categorize components as brain, muscle, heart, eye, line noise, or other categories of components. Only components classified by ICLabel as “Brain” or “Other” with above 42% certainty were preserved in the data (on average, 19 components were filtered per subject). The data used in the ERP analyses was separately high- and low-pass filtered using a low cutoff of 0.1 Hz (Kaiser window, maximum passband deviation: 0.001 and transition bandwidth: 0.2 Hz) and high cutoff of 17 Hz (Kaiser window, maximum passband deviation: 0.0001 and transition bandwidth: 4.25 Hz) using the EEGLAB function “pop_firws” (v2.8; Widmann et al., 2015). Data used for other analyses were left unfiltered. Epochs were cut from −1,500 to 1,500 ms around search stimuli onset with a specific window of interest of −500 to 500 ms around search onset. Epochs that exceeded the adaptive channel threshold within this time window of interest were discarded. Furthermore, epochs containing an eye movement (threshold of 30 μV [∼ 1.5°]) or a blink (identified by an adaptive threshold procedure) in this 1,000 ms window of interest were discarded. As a final preprocessing step, data were transformed to reference-free current source densities by computing the surface Laplacian to focus on high-spatial-frequency components and reduce volume-conducted potentials or distributed sources (Perrin et al., 1989).

### Frequency analyses

SSVEP analyses were restricted to the frequencies of the two horizontal locations of interest, as these two locations could be the high-probability distractor location across the two sessions. We additionally restricted our distractor-tuned analysis to search trials in which the distractor was at a horizontal location and the target was at a vertical location. For our target-tuned analysis, we restricted distractors to the vertical locations or absent and the target was horizontal. The window of interest for analysis was selected to be −500 to 500 ms surrounding search array onset. To quantify SSVEP modulations across time, activity from −1,500 to 1,500 ms centered on stimulus presentation was transformed to the frequency domain using fast Fourier transform. This data was then convolved in four separate analyses with spectral Gabor kernel centered at the driving frequency of interest (FOI) with a half-band filter width of 1.4 Hz (a different FOI was used on each step, producing separate time courses for each frequency). Finally, the convolved data was then inverse Fourier transformed back to the temporal domain to obtain a time-resolved frequency-tagged response. The Gabor kernels were constructed using the following formula:

$$\exp\left(-{\left(\frac{\;f-\mathrm{FOI}}{2\sigma }\right)}^{2}\right),\;(1)$$

where *f* is the frequency vector containing the specific frequency bins at which the Fourier transform evaluated the signal, FOI is the center of the frequency of interest, and sigma is the standard deviation calculated as follows:

$$\sigma =\frac{\mathrm{HalfBandWidth}}{\sqrt{2\log(2)\;}}.\;(2)$$

The real and imaginary components of the data were then converted into magnitude by taking the absolute value of the signal, thereby coding for the dynamic signal amplitude. The full width at half maximum was calculated to determine the temporal fidelity of our outcomes using the following formula:

$$\frac{\log(2)\;}{\pi \cdot \mathrm{HalfBandwidth}}.\;(3)$$

With a half band width of 1.4 Hz, our Gabor analysis had a temporal integration window of roughly 160 ms. An automated electrode selection procedure was used to select the electrodes of interest in our subsequent analysis (Ma et al., 2019; Zhigalov et al., 2019). This selection procedure chose the two electrodes with the highest tagged response from the contralateral hemifield to the tagged stimuli while search stimuli were on screen. Importantly, this selection was merely based on the overall tagged response, agnostic to whether it was an HP or LP location (if, for instance, selection was based on the greatest difference between HP and LP conditions, then it would be an example of double dipping. Instead, selection only guaranteed the strongest signal was compared in each condition). Selection was restricted to the 21 posterior electrodes which provided broad coverage of the visual areas of interest (Fig. 3*A*, blue dots). Selection was further based on the combined average of search trials in which either a distractor or target was at the location of interest to isolate the steady state when attention was engaged by a salient item (Fig. 3*A* for the total electrode selection density). Note that this was done separately for each individual and session.

Our main analysis was split into two windows of interest—the presearch period and the post search period probing proactive and reactive mechanisms, respectively. For our proactive analysis, we needed a baseline-free measure, because we were interested in the tagged response in the presearch window. We therefore used a normalization index (Zhigalov et al., 2019; van Moorselaar et al., 2020; Zhigalov and Jensen, 2020; Duncan et al., 2023a) to compare the amplitude of tagged responses when a location was a high-probability distractor location versus when it was a low-probability location across the two experimental sessions. The normalized score was calculated as follows:

$$\frac{HP-LP}{HP+LP}.\;(4)$$

Traditionally, normalization indices compare responses between brain hemispheres within the same set of trials. While this works well for analyzing endogenous oscillations (e.g., alpha) where the same frequency is analyzed in both hemispheres, it becomes problematic when using different frequencies in each hemifield, as each tagging frequency has its own baseline responsivity that varies across participants (Fig. 3*B*, noting the general downward slope as frequencies increase). We therefore calculated a normalized score across sessions instead.

This normalization approach calculates the relative contribution of each condition (HP and LP) to the total measured signal (HP + LP). A positive value indicates a stronger tagging response was recorded when that location was a high-probability distractor location and a negative value would indicate the opposite. If both conditions contributed equally, the value would be 0. The normalized value represents a percentage—for example, a value of 0.1 indicates that the HP condition contributed 10% more of the total signal than the LP condition.

This approach allowed us to compare two conditions in the same hemisphere using the same underlying frequency tag, but across sessions. Importantly, this measure does not require baselining, enabling comparison of the total ongoing signal during what would normally be the baseline period. Furthermore, since this measure is taken before search onset, all trials were collapsed together without regard to the upcoming search condition for this analysis. This further ensured ample and matched trial counts across our proactive analyses.

In addition to signal amplitude analysis, based on a discrepancy with results from the Ferrante et al. study, we also conducted an analysis based on signal coherence. Other work examining proactive modulations in tagged responses have not focused on the magnitude of the complex component of the fitted tagged signal as above, but instead on its correlation of the observed signal with the pure signal (Ferrante et al., 2023). We chose to also analyze coherence to observe the fit of the observed signal with the stimulation using the intersite phase-clustering (ISPC) approach (Lachaux et al., 1999; Penny et al., 2008; Cohen, 2014). To do this, the data was first filtered with a fourth-order Butterworth zero-phase filter with range 1.4 Hz above and below each frequency of interest. The data was next transformed into instantaneous phase (*Φ*) and magnitude (*M*) using a Hilbert transform. The transformed data was then restricted to our window of interest (−500 to 0 ms prior to search onset). ISPC scores were calculated as follows:

$$\left|n^{-1}\underset{t=1}{\sum }\;e^{i(\phi_{xt}-\phi_{yt})}\right|,\;(5)$$

with *Φ*_xt_ and *Φ*_yt_ representing the instantaneous phase for the real and reference signals at each timepoint, respectively, and *n* representing the length of the time window of interest. ISPC coherence in the prestimulus period was then averaged for HP and LP locations per participant and session in the preidentified electrodes of interest. Modulation as a result of statistical learning was compared using the same across-session normalization approach described above to produce a single number comparing relative coherence at suppressed and nonsuppressed locations in space ding the presearch period.

A further post hoc analysis of alpha was carried out to determine whether modulations of alpha lateralization in the same prestimulus period would be observed. This was done using the same analysis pipeline as the SSVEP approach, using nine overlapping kernels centered at 0.5 Hz intervals between 8 and 12 Hz. Furthermore, rather than iteratively selecting the electrodes of interest via an automated procedure, standard occipital electrodes were used in accordance with previous work (PO4/5, PO7/8, O1/2; Wang et al., 2019b). These extracted frequency responses were subject to the same across-session normalization to compare within hemispheric alpha when a distractor was expected in that hemifield versus when it was not expected, across sessions. These normalization scores were then averaged to get an overall time course of alpha lateralization in the presearch period which as subsequently binned in the −500 to −200 ms window to index proactive lateralization.

For post search analysis, after averaging across the electrodes of interest, the tagged response was baselined in the −500 to −200 ms window prior to search onset using division. Importantly, HP and LP conditions had different trial counts, each SSVEP condition was subsampled 5,000 times by selecting a random subset of samples from the larger HP condition matching the number of trials in the smaller LP condition. This was done to equal signal variance in the baseline window as smaller trial counts were found to reliably result in larger overall amplitudes, translating to larger baseline values (and thus more drastic normalization). The average tagged response was calculated for each subsample and then averaged over the 5,000 subsamples to get a group average based on matched trial counts. Participant data was then averaged across the two sessions, giving an HP and LP time course which was hemisphere- and tagging-frequency neutral.

### ERP analysis

Although alternative accounts have been proposed, many studies have linked the distractor positivity (P_D_) to distractor suppression (Hickey et al., 2009; Gaspelin et al., 2023; but van Moorselaar et al., 2023; Oxner et al., 2024). This component, which is defined as a positive deflection in the PO7/8 electrode contralateral to a visual distractor, can occur in the time range between 100 and 500 ms, and this variable latency has been used to dissociate between proactive and reactive suppression mechanisms. Whereas relatively late P_D_ values are thought to index a suppressive process involved in recovery from capture (Hickey et al., 2009; Sawaki and Luck, 2013), relatively early P_D_values have been proposed to signal efficient distractor rejection, possibly driven by proactive mechanisms that preemptively prevents attentional allocation toward a salient stimulus (Weaver et al., 2017; Wang et al., 2019b; Taylor and Feldmann-Wüstefeld, 2024 for a review of both components, Gaspelin et al., 2023). Given that we were interested in examining how statistical learning shapes the temporal dynamics of distractor processing, we examined lateralized distractor positivity values in a broad window of interest (see window selection procedure below).

Epochs were baseline corrected by subtracting the mean signal in the −200 to 0 ms prestimulus baseline period from all timepoints. To enable isolation of lateralized distractor-specific components, we focused on search trials in which the distractor was presented on left or right horizontal locations, while the target was on the vertical meridian (Hickey et al., 2009). Trials were further split into HP and LP conditions depending on where the high-probability distractor location was on that session. Waveforms evoked by visual targets or distractors were collapsed across left and right electrodes by flipping the sensor topography when stimuli of interest were presented in the right hemifield (i.e., PO7 became PO8 when relevant stimuli were presented on the left side of the screen) to produce aligned waveforms for contralateral and ipsilateral scalp regions. Lateralized difference waveforms were then computed by subtracting the ipsilateral waveform from the corresponding contralateral waveform.

Condition peaks were calculated across HP and LP conditions (note that separating the analysis to use separate condition peaks did not change the pattern of results). Windows were centered around the peak amplitude difference (±25 ms) in distractor-tuned analyses in the early P_D_ window (i.e., 0–200 ms) and late P_D_ window (i.e., 200–500 ms). As a results, voltages were analyzed in 50 ms windows centered at ∼145 ms for the early P_D_ window and 299 ms for the late P_D_ window. In addition, differences in onset latency were evaluated with a jackknife procedure (Miller et al., 1998; Kiesel et al., 2008), with the window of interest being the first 200 ms following search onset and onset latency being measured as the earliest timepoint where the component of interest reached 50% of its peak amplitude.

### Linear mixed effects analysis

To test the influence of intertrial priming, we conducted a linear mixed effects analysis (Bates et al., 2015). RTs were designated as the dependent variable with two fixed effects included in the starting model. These effects were distractor condition (distractor absent or present at high or low probability locations) as well as trial repetitions (repetition yes/no). We built our model by first including only the first repetition of trials and adding additional repetitions until the added effects no longer improved the model fit. The random effect's structure included a by-subject random intercept, but slopes were kept constant across fixed effects as including these slopes induced singular fits. The distractor location factor was set at default to the HP trials. For repetitions, the default was no repetition. The best performing model formula was as follows:

$$\begin{array}{rl} \mathrm{model} & =rt\;∼\;\mathrm{distractor}\_\mathrm{conditio}n_{\mathrm{none}/\mathrm{high}/\mathrm{low}}\\ & \;+\mathrm{distractor}\_\mathrm{repetition}\_1\mathrm{bac}k_{\mathrm{yes}/\mathrm{no}}\\ & \;+\mathrm{distractor}\_\mathrm{repetition}\_2b\mathrm{ac}k_{\mathrm{yes}/\mathrm{no}}+(1|\mathrm{subjec}t_{\mathrm{id}}). \end{array}$$

All other features of the linear model used the default settings from the lmer package in r (lme4 package version 1.1.35.3). The model had 60,000 observations and a log-likelihood of −410,866.4.

### Statistics

Behavioral results were first analyzed using repeated-measures analyses of variance (rANOVAs) and followed up with planned paired two-tailed *t* tests. SSVEP time courses were analyzed using cluster-based permutation tests and paired two-sided *t* tests with cluster correction (*p* = 0.05 and 1,024 iterations) using MNE functionality (Gramfort et al., 2014). For our proactive analyses, as well as the analyses of preidentified ERP components, summed neural activity was analyzed by binning the average activity per participant in the window of interest and compared with paired-sample two-tailed *t* tests. The windows of interest were identified in different ways depending on the analysis. For the analysis of proactive SSVEP amplitude, our window of interest was −500 to −200 ms prior to search onset, thereby matching the baseline window in our reactive analysis. For our ISPC Coherence analysis, the analysis itself does not produce a measure for each timepoint, but a general coherence score calculated over a slice of data over time. For this reason, a larger window was necessary to increase data density, and so the window from −500 to 0 ms preceding search onset was used.

## Results

### Behavior

Of the 24 participants, 20 reported noticing that targets and distractors only appeared at the four cardinal locations, and six reported noticing the distractor regularity. Of these six, only two were then able to report the distractor high-probability location within the last session. Excluding these participants did not significantly alter the patterns of results. Due to their low number, awareness was not treated as a variable of interest in all subsequent analyses.

It is well known that when targets and distractors are presented at the same location consecutively this will result in faster response times (Maljkovic and Nakayama, 1994; Goschy et al., 2014), a condition which could influence our results due to the imbalance in trial conditions. Indeed, stimulus repetitions also speeded overall response times within the present data (*t*_(23)_ = 5.781, *p* < 0.001, *d* = 1.18). To control for this effect, distractor repetitions were excluded. Further exploration of the data revealed that participants responded significantly faster when distractors were presented along the horizontal midline than vertically (*t*_(23)_ = 2.845, *p* = 0.009, *d* = 0.581). This horizontal bias is a well-known phenomenon (Abrams et al., 2012) which could influence our behavioral analysis as the HP locations could only be on the horizontal axis and therefore are expected to be faster/better processed than vertical stimuli. We therefore also restricted our analysis to trials where the distractor was presented at one of the horizontal locations (note that this resulted in our behavioral analyses matching the same subsets of trials as used in our analyses of evoked neural signals).

**Figure 2.**
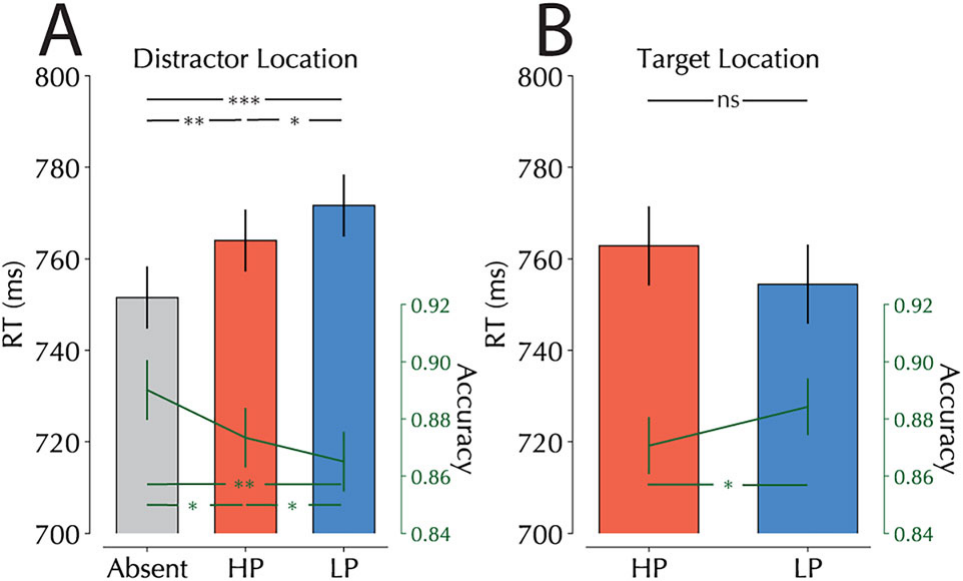
Reaction times and accuracies separated for distractor and target conditions. ***A***, Reaction times and accuracy as a function of distractor location and presence. Bars represent reaction times, green lines represent accuracy. Error bars represent within-subject 95% confidence intervals (Cousineau, 2005; Morey, 2008). Note that this figure excludes trials in which the distractor was repeated in two consecutive trials and is also restricted to trials in which the distractor was presented at one of the horizontal locations (matching the trials used in our neural analysis). ***B***, Reaction times and accuracy as a function of target location. Bars again represent reaction times while green lines represent accuracy and error bars represent within-subject 95% confidence intervals. Trials in this analysis were restricted to those in which the target was present at one of the horizontal locations and excludes target repetition trials. Note that the top significance stars only refer to reaction time data while the bottom significance stars refer to accuracy for both figures (*p < 0.05; **p < 0.01; ***p < 0.001).

An rANOVA taking distractor condition (absent, LP, and HP) as a factor and reaction times as the dependent variable found a significant main effect of distractor conditions (*F*_(2,46)_ = 21.1, *p* < 0.001, *η*^2^ = 0.006; Fig. 2*A*). Further pairwise testing showed a reliable slowing when a distractor was present (*t*_(23)_ = 6.4, *p* < 0.001, *d* = 1.306), replicating the standard additional singleton effect in an SSVEP setup (Theeuwes, 1992). Furthermore, we observed a significant difference between reaction time cost in HP and LP distractor conditions, with costs being smaller in the HP condition (*t*_(23)_ = 2.292, *p* = 0.031, *d* = 0.468), thereby replicating the standard statistical learning effect (Wang and Theeuwes, 2018). The same pattern of results was with overall accuracy, where the main effect of distractor condition (*F*_(2,46)_ = 8.384, *p* = 0.001, *η*^2^ = 0.022) again reflected smaller costs at HP relative to LP conditions (*t*_(23)_ = 2.389, *p* = 0.025, *d* = 0.488).

To further examine the influence of distractor location regularities on spatial suppression, we tuned the previous analysis to target locations. While numerically, there was a hint that target processing, as observed previously (Ferrante et al., 2018; Sauter et al., 2018; Wang et al., 2019a), was impaired at high-probability locations, this effect was not reliable in response times (*t*_(23)_ = 1.454, *p* = 0.159; Fig. 2*B*). However, target detection was reliably less accurate at high-probability distractor locations (*t*_(23)_ = 2.155, *p* = 0.042, *d* = 0.44). Together these behavioral findings demonstrate clear evidence of learned spatial suppression in a paradigm where stimuli were constantly flickering on the screen.

As a further exploratory analysis, a linear mixed effect approach was used to disentangle the relative contribution of intertrial priming and statistical learning (van Moorselaar et al., 2021; Duncan et al., 2023a). Removing repetition trials as above is one accepted method of controlling for these short-term priming effects (Goschy et al., 2014, Sauter et al., 2018, supplementary discussion), but a more inclusive approach is using linear mixed models, which allow for the inclusion of multiple effect structures. This approach allows to determine the relative contribution of multiple effects within one go without excluding trials. In essence, it makes it possible to observe statistical learning effects while simultaneously controlling for intertrial priming effects. Furthermore, we can also determine how far in the past a distractor repetition continues to affect performance. We constructed our linear model by iteratively adding distractor repetitions features from further and further in the past until our model no longer benefited from the additional factors. The final model included *n*-2 trials in the past. This model showed a significant effect of distractor condition driven by statistical learning (*β*_lp_ = 9.014 ms, *β*_no distractor_ = −12.63 ms, *p* < 0.001) as well as for *n*-1 repetitions (*β*_rep_ = −12.63 ms, *p* = 0.039) and *n*-2 repetitions (*β*_rep_ = −4.614 ms, *p* = 0.021). The model no longer improved with the inclusion of *n*-3 repetitions, which only produced a minor response time decrease (*β*_rep_ = −4.272 ms, *p* = 0.055). Together, this analysis revealed distractor repetitions influenced search up to two trials in the past but that statistical learning effects continues to exist above and beyond these effects.

### SSVEP amplitudes

**Figure 3.**
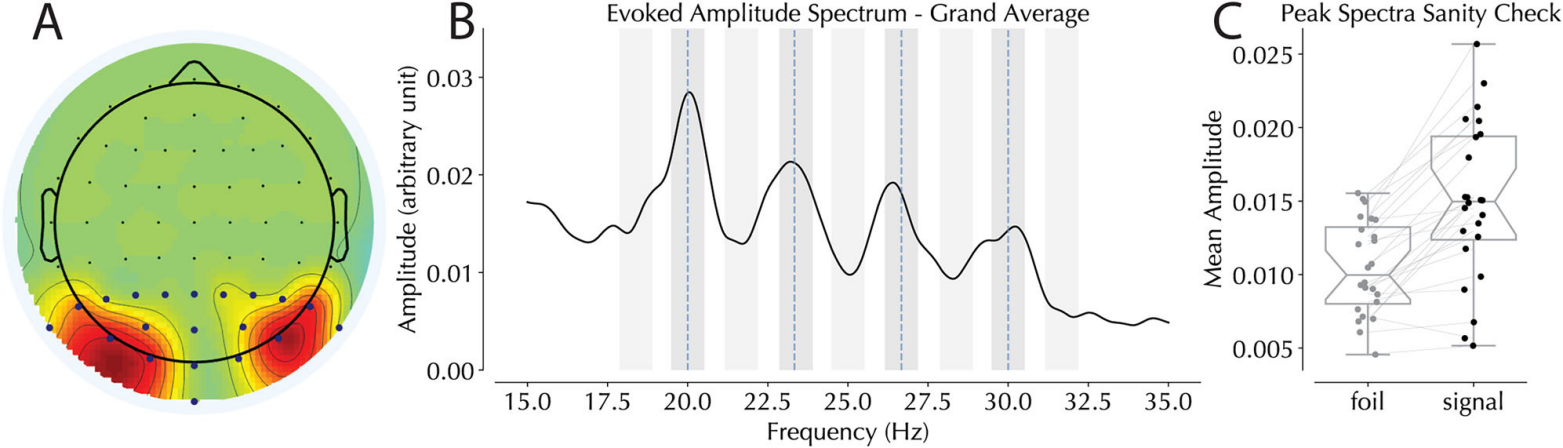
Steady-state evoked potential data. ***A***, Topography of electrode selection across all 24 subjects. Blue dots represent the 20 posterior electrodes from which electrodes of interest were selected. ***B***, Amplitude spectra recorded from the posterior electrodes with frequencies of interest represented by blue vertical dotted lines. Shaded gray areas represent bins selected in the peak spectra sanity check (darker, signal; lighter, foil). ***C***, Comparison of expected real tagged signal to intermediary foils. Note that this analysis only includes the two horizontal frequencies of interest selected per participant.

To confirm that our frequency-tagged stimuli elicited a steady-state visual evoked potential, we first analyzed the spectral power of posterior electrodes within the −500 to 500 ms window relative to search onset. This analysis was aimed at verifying the expected peaks in the frequency bands centered around the tagged frequencies (Fig. 3*B*). Visually these expected peaks were present, and to test the reliability of these peaks across participants, test bins were drawn to compare tagged responses to nontagged responses nearby in the spectra. Specifically, we sought to make a signal-to-noise test by comparing 1 Hz bins were drawn either centered on the expected driving frequencies or around foil frequencies 1.66 Hz adjacent from these frequencies of interest (foils were centered halfway between the FOIs; Fig. 3*B*, gray shaded areas). Given that these foil frequencies were drawn in between the tagged frequencies of interest, they effectively represented noise. Because spectral power is known to decrease with higher frequencies, by averaging the value in two successive foil bins per subject, a hypothetical value for the in between stimulated bin could be calculated if no frequency tagging had occurred (see Özkan and Störmer, 2024, for a similar approach). These average values could then be compared with those in the stimulated bin to estimate SSVEP signal-to-noise for each subject. Because our analysis was restricted to the two horizontal frequencies, bins were restricted to these frequencies and their adjacent foils per participant. Amplitudes were compared using paired two-tailed *t* tests and found reliable differences between signal and foil bins (*t*_(23)_ = 6.662, *p* < 0.001, *d* = 1.36; Fig. 3*C*).

**Figure 4.**
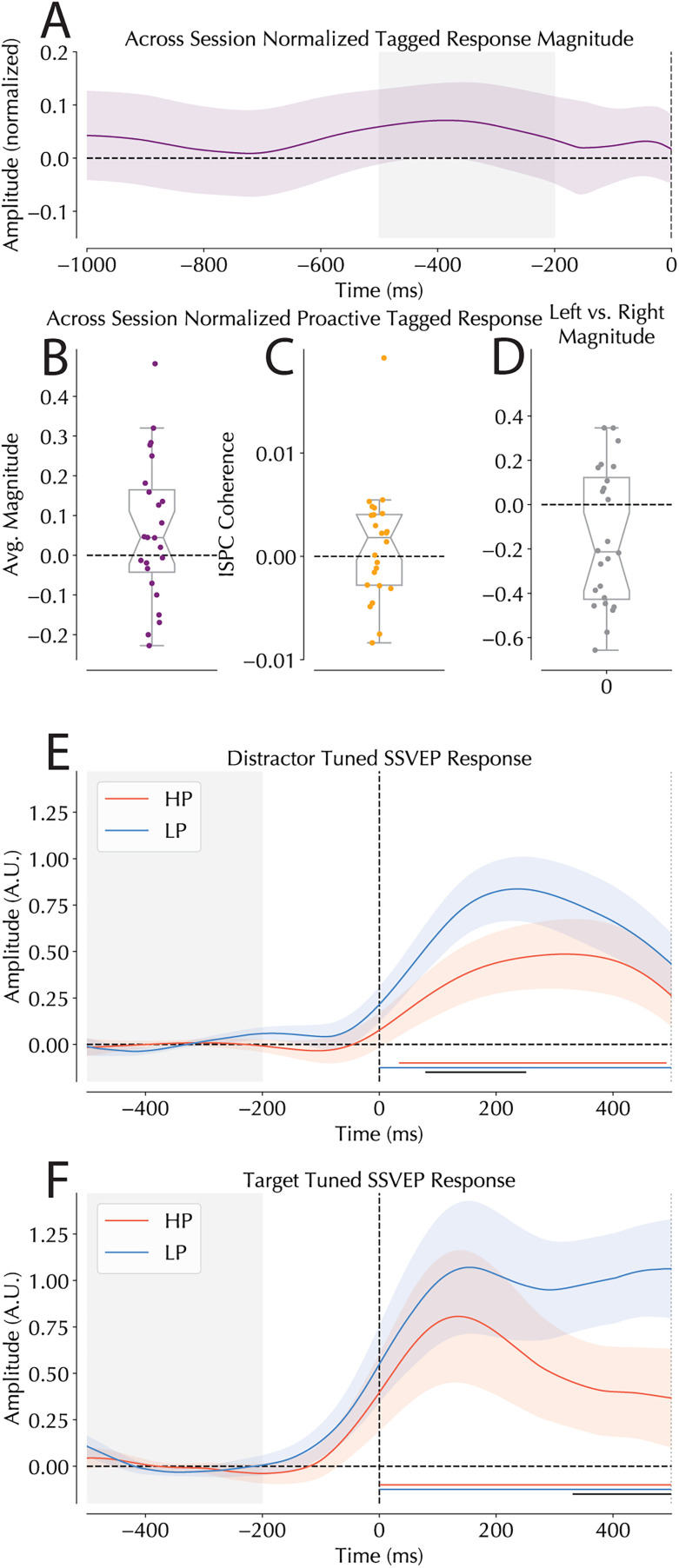
Proactive and reactive SSVEP responses. ***A***, Normalized SSVEP magnitude values in the presearch period. Shaded area represents the −500 to −200 ms window used in the binned analysis in panel ***B***. ***B***, Binned proactive amplitude modulations. Mean signal amplitude in the baseline period (−500 to −200 ms) normalized across sessions comparing HP and LP locations showed no reliable modulation in proactive tagging amplitudes. Individual dots represent single participant means, and boxplot middle indicates group median with notches indicating 95% confidence window. Box edges indicate interquartile range. ***C***, Across-session normalized coherence scores comparing HP and LP locations presearch. Average signal coherence in the 500 ms prior to search onset are compared between HP and LP locations. Similar to the magnitude-based analysis, no reliable modulation in the proactive tagged coherence was found. ***D***, Comparison of the average SSVEP magnitude in the left versus right hemisphere across sessions. Values were averaged for the entire 2,000 ms window of interest centered on search presentation. Values were calculated as left minus right, meaning that a negative value represents a stronger tagged response from the right hemisphere (note that values correspond to a percentage out of 1). ***E***, SSVEP amplitude modulations following search onset separated for HP and LP distractor trials. Gray shaded areas represent the baseline period. Colored bars represent the outcome of cluster-based permutation tests revealing significant deviations from zero for the HP (red) and LP (blue) tagged responses, indicating reliable increases in tagging amplitude following distractor presentation. Black lines represent reliable differences between HP and LP amplitudes. Shaded areas represent the within-subject 95% confidence intervals calculated using the Cousineau method (Cousineau, 2005; Morey, 2008). ***F***, SSVEP amplitude modulations following search onset for trials in which the target was presented at the HP location. Color codes match those used in the distractor figure with the shaded areas again being within-subject 95% confidence intervals.

Having confirmed the reliability of the tagged response across participants, we next observed whether our SSVEPs revealed proactive suppression of the HP distractor location. Following the work by Ferrante et al. (2023), we expected that tagged responses would decrease in the suppressed hemifield prior to stimulus onset, thereby providing convincing evidence for a proactive mechanism underlying statistically learned suppression. An across-session normalized score was calculated for each tagged frequency at the HP and LP locations which swapped across experimental sessions (see SSVEP methods). An initial cluster-based analysis of the time course revealed no significant deviations from zero (Fig. 4*A*). The tagged response was then binned in the −500 to −200 ms window (matching the baseline window in our reactive analysis). Unlike Ferrante et al. (2023), our data showed no reliable modulation of the tagged response prior to search onset (*t*_(23)_ = 1.338, *p* = 0.194; Fig. 4*B*). It should be noted, however, that the analysis approach adopted in the Ferrante et al. (2023) study focused on correlations between the observed SSVEP signal and the actual signal present on the screen. This differs from the magnitude analysis reported above, as the key variable in Ferrante et al.'s approach is not the amplitude of the matching waveform but rather its synchronicity. To replicate this analysis, we also conducted a coherence analysis to compare the synchrony of our actual and observed signals (see Materials and Methods). Using this analysis, we again observed no evidence for a proactive mechanism (Fig. 4*C*). Possible reasons for not finding proactive signal suppression are explored further in the discussion section.[Fig JN-RM-1973-24F5]

**Figure 5. JN-RM-1973-24F5:**
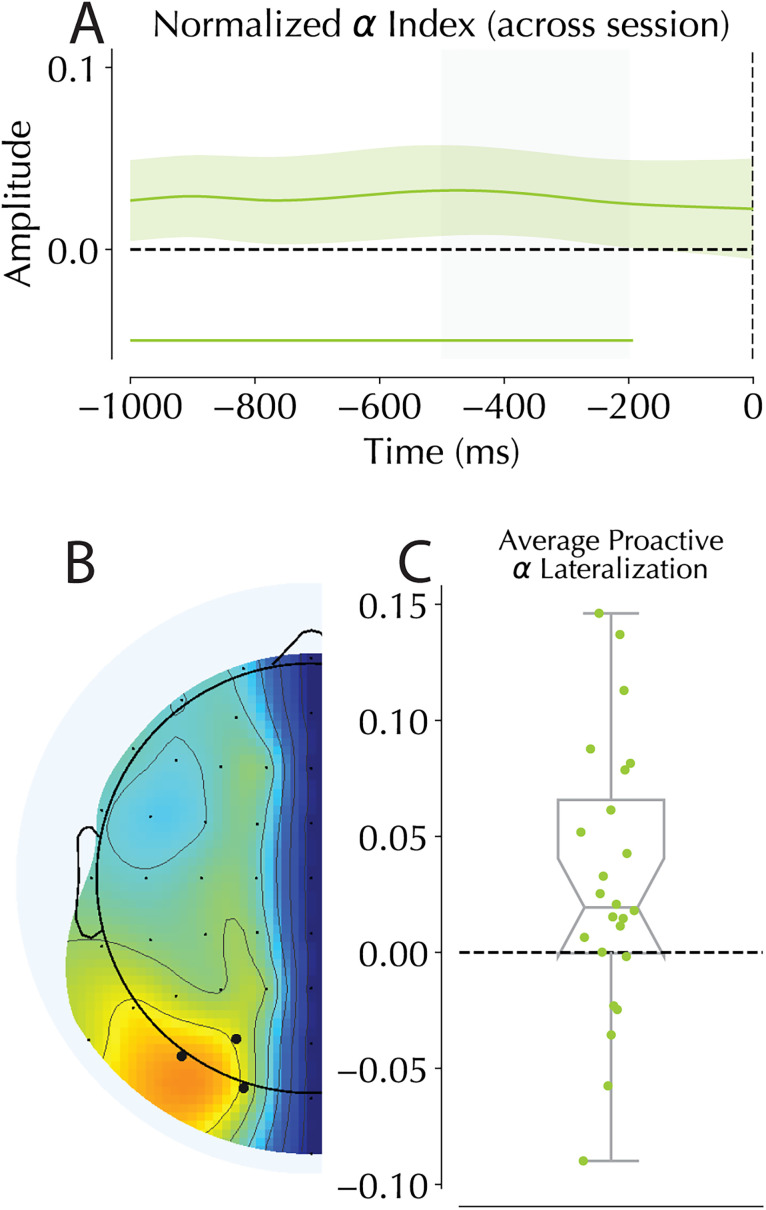
Alpha lateralization in the presearch period. ***A***, normalized time course of alpha for the 1,000 ms prior to search onset. Shading represents 95% confidence interval of the mean. Solid green bars at the bottom of the figure represent significant clusters detected by cluster-based analysis testing for differences from zero. Gray shaded area covers the −500 to −200 ms window used in panel ***C***. ***B***, Topography of alpha following averaging over our frequency bins. Black dots represent selected electrodes of interest. ***C***, Averaged alpha lateralization in the prestimulus period −500 to −300 ms prior to search onset. Scores represent HP minus LP, and thus a positive score represents more alpha in the hemisphere contralateral to the expected distractor location. Because this is a normalized score, values represent percentage differences between conditions (out of 1).

Having observed no reliable modulations in our baseline period, we proceeded to analyze the amplitude modulation of our reactive SSVEP signal. Prior to analyzing distractor and target trials, we first examined whether reliable differences in the left and right hemisphere were present. This was done by combining trials in which a target or distractor was presented laterally and averaging magnitude over the entire 2 s window of interest. We observed that participants reliably evoked a higher tagging response in the right hemisphere, replicating a result that is typically observed (Ferrante et al., 2023; Fig. 4). This, along with reliably differences in baseline tagging receptibility observable in Figure 3*B*, justified our two-session design where comparisons were confined within frequency and hemifield.

Beginning with distractors, we observed a reliable increase in the tagged response relative to baseline following stimulus onset (Fig. 4*E*). Our cluster-based analysis revealed reliable modulations of the SSVEP signal above baseline for much of the stimulus presentation window. Furthermore, and in agreement with the behavioral data, a significant difference in SSVEP amplitudes was observed between LP and HP distractor locations, indicating that distractors presented at the high-probability distractor location reliably evoked a smaller SSVEP amplitude modulation than those at LP locations (∼72 to 258 ms post search onset, though the timing of clusters should be interpreted with caution: Sassenhagen and Draschkow, 2019). Notably, this difference arose relatively early in stimulus processing, peaking ∼180 ms.

When the same analysis was yoked to targets, again a reliable amplitude modulation was observed relative to baseline with both HP and LP locations evoking a significantly enhanced response (Fig. 4*F*). Furthermore, and complementing our distractor analysis, a significant difference was observed between targets at the high- versus low-probability distractor locations. This difference arose relatively late in comparison with distractors, onsetting in the second half of stimulus presentation and growing through the remainder of the stimulus period.

### Alpha analysis

As an exploratory analysis, we also looked at alpha in the prestimulus period. While alpha's role in preparatory target processing is relatively clear (Worden et al., 2000; Foster et al., 2017), its role in distractor suppression is mixed (Noonan et al., 2016; Foster and Awh, 2019; Gundlach et al., 2020; van Zoest et al., 2021). Previous work has suggested that statistical learning may evoke a specific alpha band lateralization pattern in anticipation of search onset, with increased alpha contralateral to the high-probability distractor location (Wang et al., 2019b). However, subsequent attempts to replicate this lateralization effect failed across a number of studies with various designs (van Moorselaar et al., 2020, 2021; Ferrante et al., 2023; Qiu et al., 2023; Duncan et al., 2023a). Even though it was not a primary research question, the analysis of alpha in the current dataset showed basically the same alpha lateralization effect as was shown by Wang et al. (2019b). Similar to our SSVEP analysis, an across-session normalization score was calculated for the average of nine frequencies bins with evenly spaced centers between 8 and 12 Hz in the 1,000 ms interval prior to search onset. A cluster-based permutation test identified reliably larger alpha-power contralateral to the high-probability distractor location. Binning the normalized response within a −500 to −200 ms window prior to search onset also revealed a reliable increase in alpha contralateral to the high-probability distractor location (*t*_(23)_ = 2.504, *p* = 0.02, *d* = 0.511; Fig. 5), indicating a robust alpha effect in the current data.

### Event-related potentials

**Figure 6.**
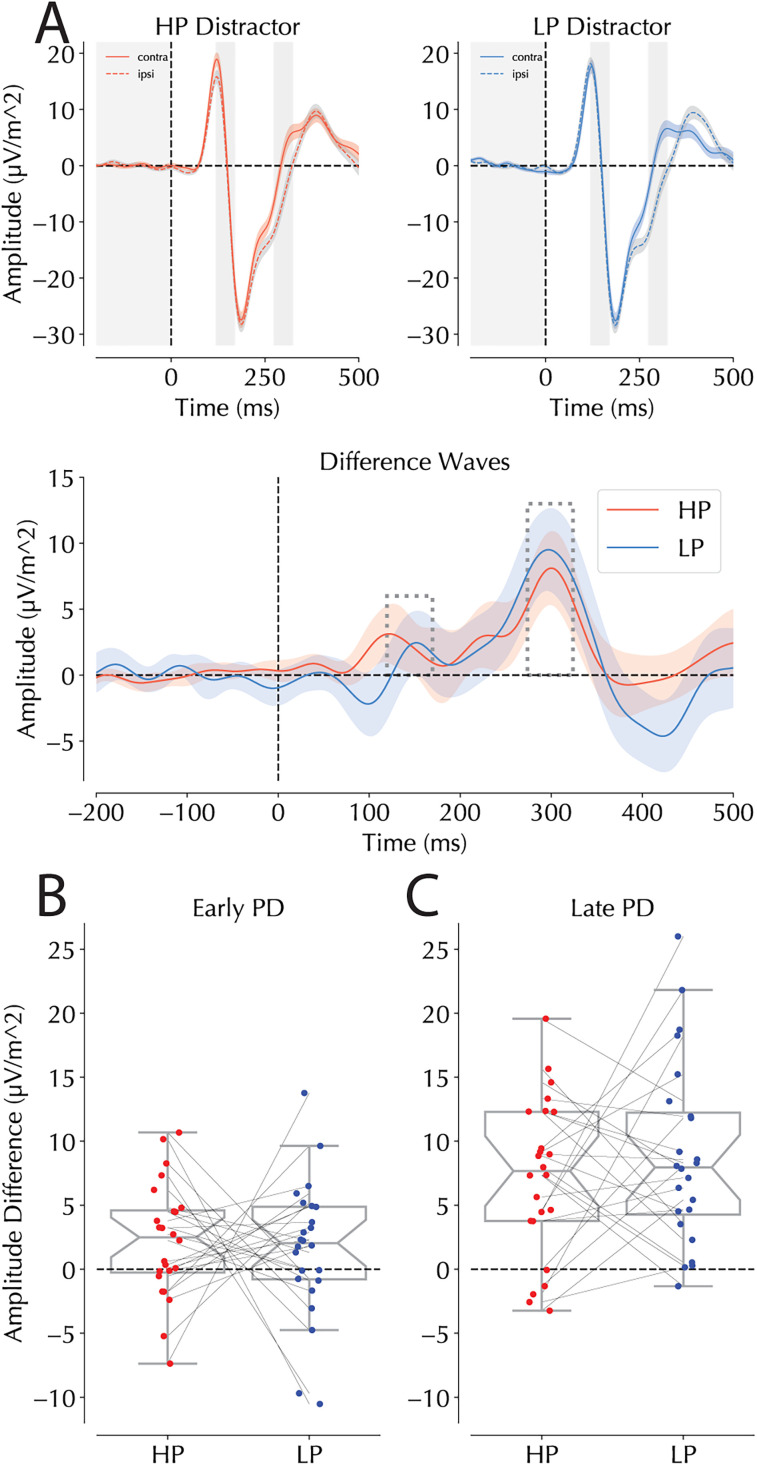
ERPs following distractor and target presentations at the HP and LP locations. ***A***, ERPs following distractor presentation at HP (red) and LP (blue) locations at PO7/8, respectively. Note that trials were restricted to those where the target was on the vertical meridian. The top panels represent condition-specific ERPs for HP (left) and LP (right) trials separating contralateral and ipsilateral sensors (dotted lines represent ipsilateral activity). The hatched shaded bars represent the windows of interest selected for the early (left) and late P_D_ (right) analyses isolated on peaks identified in HP and LP trials separately. The bottom panel shows the difference curve calculated as the contralateral-minus-ipsilateral signal across time. Colored bars represent significant deviations from zero as revealed by a cluster-based permutation test. Significant differences between the two lines would have been denoted by black bars. Shaded areas in the top two panels are within-subject corrected while those in the bottom panel are not. ***B***, Binned lateralized responses in the early P_D_ window for HP and LP trials calculated by subtracting ipsilateral form contralateral waveforms. Dots represent individual participants with lines connecting their scores across the two conditions. ***C***, Binned lateralized responses in the late P_D_ windows of interest. Boxplots in ***B*** and ***C*** follow the same conventions as Figure 4*A*.

Having established that early distractor processing differed between probability locations through SSVEP analyses, we next examined whether these location-specific attenuations were also reflected in the ERP responses to lateralized distractors. Voltages elicited by lateralized distractors within the early P_D_ interval (145 ± 25 ms) were analyzed with an rANOVA with within subjects factors Distractor Condition (HP, LP) and Hemifield (contralateral/ipsilateral to distractor) which yielded a main effect of Hemifield (*F*_(1,23)_ = 33, *p* < 0.001, *η*^2^ = 0.093) that was not accompanied by an interaction (*F*_(1,23)_ = 1.63, *p* = 0.69; Fig. 6*B*). However, a subsequent jackknife procedure confirmed the earlier onset of the positivity in the HP condition (*Δ_onset_* = 19.9 ms; *t*_(23)_ = 3.718, *p* < 0.01). This pattern of results suggests that despite the clear difference in latency, the amplitude of the elicited positivity did not differ across conditions. For the late P_D_ window (299 ± 25 ms), the main effect of Hemifield (*F*_(1,23)_ = 71.74, *p* < 0.001, *η*^2^ = 0.537) was again not accompanied by an interaction (*F*_(1,23)_ = 1.27, *p* = 0.283; Fig. 6*C*). For the late P_D_, no modulation in timing was observed (*t* < 1).

## Discussion

The present study investigated the temporal neural dynamics of learned distractor suppression by combining frequency tagging with a probabilistic variant of the additional singleton paradigm (Wang and Theeuwes, 2018). This novel methodological approach integrated SSVEPs—which provide continuous measurement of attentional processing with excellent temporal resolution—with the P_D_ component, a putative neural marker of suppression. While SSVEPs have recently emerged as a powerful tool for studying attentional processing in visual search (Forschack et al., 2022a,b; Ferrante et al., 2023; Bouwkamp et al., 2024; Duecker et al., 2024), our study represents the first application of this technique to parallel search involving a salient target and distractor using the additional singleton paradigm. Despite the continuous flicker of display elements, we replicated classic behavioral markers of statistical learning: reduced distractor interference at high-probability compared with low-probability locations (Goschy et al., 2014; Ferrante et al., 2018; Wang and Theeuwes, 2018). Crucially, this behavioral signature of learned suppression was accompanied by two distinct neural effects: attenuated SSVEP responses to distractors at high-probability locations and accelerated early P_D_ onset. Yet, we observed no anticipatory modulation in the SSVEP signal, suggesting that suppression was not anticipatory but only kicked in following stimulus onset.

The constant flickering of placeholders and search stimuli allowed us to track the effect of the spatial distractor imbalance at distinct moments in time: in anticipation of the search display and in response following search display onset. First, examining the prestimulus period, we found no reliable modulation of baseline SSVEP signals at high-probability (HP) compared with low-probability (LP) locations, using either magnitude or coherence measures. This absence of anticipatory modulation is inconsistent with recent findings (Ferrante et al., 2023) and argues against suppression operating before display onset. However, we did find enhanced lateralized alpha activity in HP trials, replicating a previous finding and suggesting some preparatory processing (Wang et al., 2019b). Furthermore, immediately following display onset, we observed clear evidence of differential processing across probability locations. In line with recent fMRI evidence of reduced BOLD responses at suppressed locations (Won et al., 2020; Adam and Serences, 2021; Zhang et al., 2022; Richter et al., 2024), SSVEP response to HP distractors was significantly attenuated compared with LP distractors, with this difference peaking ∼180 ms poststimulus.

Following search array onset, our SSVEP and ERP signals gave clear indications of improved suppression on HP trials, begging the critical question: does this suppression occur before the first shift of attention, qualifying it as proactive under broader definitions (Liesefeld et al., 2024), or does it reflect ultra-fast reactive disengagement? Although temporal markers of attentional capture show considerable variability, making it challenging to definitively label effects as proactive or reactive, several aspects of our data suggest an early, proactive mechanism operating before the first shift of attention to the distractor. First, at the behavioral level the effect was not restricted to distractors but also affected targets that happened to appear at high-probability distractor locations. Because the target was also suppressed, it suggests that suppression kicked in early, at least before the item presented at the suppressed location was identified as being the target. Second, the modulation in stimulus evoked SSVEP amplitude emerged remarkably early, appearing to begin before 100 ms poststimulus onset—a timeframe typically associated with initial feedforward visual processing rather than attentional reorienting (Lamme and Roelfsema, 2000). This early timing also aligns with our observation of accelerated early P_D_ at HP locations, suggesting that statistical learning modifies initial stimulus processing rather than subsequent attentional operations. Importantly however, SSVEP signals remained above baseline in both conditions, indicating that while suppression attenuated distractor processing, it did not prevent it entirely, demonstrating that the learned suppression was insufficient to cancel out distractor processing altogether (note that this observation is consistent with the behavioral findings that show the distractor when presented at the suppressed location still causes reliable interference; Fig. 2*A*). Furthermore, recent work has suggested that learned suppression leads to two key phenomena: reduced salience signals from the HP location and fewer capture episodes by distractors at those locations (Duncan et al., 2024). The divergence in tagged responses could thus reflect either this reduced saliency signaling or the diminished capture rate. The early onset of this effect is more consistent with reduced saliency signaling, though the relatively large integration window of SSVEPs suggests the signal likely captures elements of both processes.

While our findings primarily support an early, potentially proactive suppression mechanism, it is important to acknowledge that proactive and reactive mechanisms are not mutually exclusive (Wang et al., 2019a; Liesefeld et al., 2024). Indeed, our data revealed both early and late modulatory effects, suggesting the operation of multiple temporal mechanisms. The early differences in distractor-evoked SSVEP responses likely reflect proactive suppression, but our target-based analysis revealed a distinct pattern that points to additional reactive components. Specifically, while distractor-related amplitude differences emerged rapidly, target-evoked SSVEP responses at high- versus low-probability locations only diverged in the latter half of the stimulus presentation period (beyond 250 ms poststimulus onset). The amplitude differences between these conditions closely aligned with performance, showing a lower tagged response in conditions where target processing was impaired.

As noted earlier, many neuroimaging studies on statistically learned distractor suppression have utilized the variable latency of the P_D_ to dissociate between proactive and reactive mechanisms. While the magnitude of the early P_D_ has been directly linked to suppression, with larger positives reflecting more efficient suppression (Gaspelin and Luck, 2018; Feldmann-Wüstefeld and Vogel, 2019), the opposite pattern has been observed for later positivities—an attenuation of the reactive P_D_ under conditions of more efficient suppression (van Moorselaar and Slagter, 2019; Heuer and Schubö, 2020; van Moorselaar et al., 2020; Abbasi et al., 2023; Hanne et al., 2024). In our study, rather than an amplitude modulation, the behavioral benefit for distractors at HP locations was accompanied by an earlier onset of the early P_D_, without a modulation of the late P_D_ component. The interpretation of this onset modulation requires careful consideration of competing theoretical frameworks. While the early P_D_ is often used to establish proactive suppression (Gaspelin and Luck, 2018; Wang et al., 2019b; van Moorselaar et al., 2021), others have proposed it reflects saliency processing, with no direct link to distraction beyond serving as a precursor to rejection (often termed the positivity posterior contralateral or Ppc; Corriveau et al., 2012; Fortier-Gauthier et al., 2012; Kerzel and Huynh Cong, 2022). Thus, one could interpret this speeded early P_D_ as reflecting either more efficient suppression or, alternatively, changes in saliency processing. However, it is important to note that other work has established that ERPs are altered by the presence of placeholders as used in our experiment (Forschack et al., 2022a); thus more work is called for to determine the generalizability of our results to non-SSVEP setups.

A recently proposed mechanism for statistical learning leans on changes to the underlying connectivity of neurons in the visual system to facilitate the behavioral effects of learning via an “activity-silent” mechanism (van Moorselaar et al., 2020; Ferrante et al., 2023; Duncan et al., 2023b; Huang et al., 2025). This “synaptic” account expects that no markers of suppression should be present prestimulus as the benefits are not mediated by active neural firings, but by changes to the responsivity of the neural ensembles coding attentional priority as a hidden layer of the combined attentional priority map. Under these assumptions, early modulations in evoked responses are expected as learned suppression should not be present in the neural signal unless evoked by some external event. SSVEPs offer an intriguing possibility to reveal these otherwise hidden biases as they constantly excite the visual system, creating a state where Ferrante et al. claimed otherwise invisible changes in underlying neural excitability were revealed (Ferrante et al., 2023). While Ferrante et al. observed a clear modulation in SSVEP coherence prior to stimulus onset, they observed no accompanying modulation in alpha. It is thus worthwhile to consider why the anticipatory modulation as observed in the study by Ferrante et al. (2023) was absent here, while anticipatory alpha responses were present.

Firstly, in regard to anticipatory SSVEP coherence, the most obvious culprit is that Ferrante et al. (2023) utilized RIFT, a form of frequency tagging where very high frequencies are used for the frequency entrainment (above 60 Hz) which results in the disguising of the flickering stimuli as static and solid (Spaak et al., 2024). Given RIFT is a relatively young method in comparison with slower, overt frequency tagging approaches that have existed for decades (Morgan et al., 1996; Müller et al., 1998), it remains largely unexplored how some results are observable using one approach and not the other. As such, the current results represent one of the first observed incongruencies between these techniques. Alternatively, the task design itself differed in several key aspects between our eight shape displays and the four shape displays used by Ferrante et al. (2023). Ferrante et al. used a high-probability hemifield while we used a high-probability location, and we presented the distractor at the high-probability location on 50% of distractor present trials, whereas their high-probability hemifield held a distractor on 75% of trials. Furthermore, a set intertrial interval was used in the design of Ferrante et al. (2023), leading perhaps to temporal preparation effects which may have influenced their clear proactive signal. These seemingly slight differences in the design could have led to participants adopting distinct strategies in approaching the task, which would have resulted in divergent encephalographic signatures.

While one of the first EEG studies investigating encephalographic markers of distractor learning reported alpha lateralization (Wang et al., 2019b), several subsequent studies, including Ferrante et al. (2023), have failed to find similar prestimulus lateralization (van Moorselaar et al., 2020, 2021; Ferrante et al., 2023; Qiu et al., 2023; Duncan et al., 2023a). Given these discrepancies, it is worth considering what features of the current study might have contributed to finding alpha lateralization contralateral to the high-probability distractor location. Two design decisions warrant special consideration: first, both the current design and that used by Wang et al. (2019b) utilized an experimental design where targets and distractors were only presented at the four cardinal locations (top, bottom, left, and right). This was done to increase the number of trials in which the distractor was lateral. While these were the only trials used in the analysis, it also resulted in a lower variability in the locations in which target and distractors were presented. This lower variability may then have compounded the influence of the second point: where both the current design and that used by Wang et al. (2019b) did not have perfectly balanced target distributions. In both designs, targets were slightly less likely to appear at the suppressed location than the other three possible locations. Again, this was done again to increase the number of trials in which the distractor was presented at the horizontal location. [Note, however, that in a design utilizing a target regularity, Duncan et al. (2023a) did not observe alpha lateralization.]

With these caveats considered, two current theories about alpha oscillations and their meaning may help explain our results. First, it has been observed that alpha signals increase in fidelity with task difficulty, making cognitive load a strong predictor of whether alpha mediated effects will be observed (Gutteling et al., 2022; Jensen, 2024). This has led to a recent proposal arguing that alpha effects are only observed in tasks that require sufficient effort (Bonnefond and Jensen, 2024). Previous studies which have investigated alpha modulations in statistical learning have used designs with empty screens in between trials during which participants are not engaged in any task (van Moorselaar et al., 2020, 2021; Qiu et al., 2023; Duncan et al., 2023a) or rapidly flickered stimuli whose flicker was imperceptible (Ferrante et al., 2023). Alternatively, the relatively short display time of the search display (500 ms) may have increased cognitive demands compared with previous studies. Second, it has also been proposed that increases in alpha contralateral to expected distractor locations requires both target and distractor predictions to be present (Bonnefond and Jensen, 2024). Therefore, the inclusion of a minor target regularity in combination with the stronger distractor regularity, as in Wang et al. (2019), may have contributed to the current results. It should be noted that these are speculations about why the current findings are different from previous ones, and more research is needed to understand the conditions under which contralateral alpha buildup occurs.

In summary, the current study combined the additional singleton task with imbalanced distractor presentations with an SSVEP design. We demonstrate early modulations in visual processing of stimuli presented at the location of learned suppression as evident by reduced SSVEP evoked amplitude changes and earlier onsets of the early P_D_ in response to distractors presented at these locations. Furthermore, we observed a proactive increase in contralateral alpha prior to search onset. Together these results indicate statistically learned suppression affects the early orientation of attention toward items in suppressed regions of space.
